# Bibliometric analysis of hypospadias from 1998–2023

**DOI:** 10.3389/fsurg.2025.1511055

**Published:** 2025-04-15

**Authors:** Dezhi Su, Xiaowei Li, Sha Li, Kun Wang, Gangquan Wu, Guomin Zhai

**Affiliations:** ^1^ Department of Pediatric Surgery, Dongguan Maternal and Child Health Hospital, Dongguan, Guangdong, China; ^2^ Department of Rehabilitation Medicine, Fifth Hospital in Wuhan, Wuhan, Hubei, China

**Keywords:** hypospadias, bibliometric analysis, bibliometrix R package, VOSviewer, CiteSpace

## Abstract

**Introduction:**

To analyze the development trends and research hotspots in the field of hypospadias through bibliometric analysis.

**Methods:**

Utilizing three bibliometric mapping tools-bibliometrix R package, VOSviewer, and CiteSpace, we summarized the overview information of the retrieved literature. We conducted analyses on the co-occurrence and development trends of authors, institutions, countries, and keywords.

**Results:**

A total of 3,647 hypospadias-related publications were included, with contributions from 14,633 authors. The keyword co-occurrence network identified seven clusters related to the diagnosis, pathophysiology, and treatment of hypospadias. Researchers have primarily focused on the genetic mechanisms of hypospadias, the role of environmental factors, surgical outcomes, anesthesia techniques, and patient satisfaction post-surgery. Emerging research hotspots include the precise selection of surgical flap sources, genomic alterations, and the correlation with syndromes related to disorders of sex development, all of which show strong potential for future breakthroughs.

**Discussion:**

Bibliometric analysis provides a comprehensive understanding of the research hotspots and development trends in hypospadias, thereby enriching the field of hypospadias research.

## 1 Introduction

Hypospadias is a common birth defect in boys that affects the genitals and is characterized by an abnormal positioning of the urethral opening due to atypical development of the urethral corpus spongiosum (1). Hypospadias often coexists with other genital abnormalities such as penile curvature and irregular penile skin. In addition to its anatomical implications, hypospadias significantly impacts the patient's physiological and mental well-being. Epidemiological studies indicate that hypospadias is one of the most prevalent congenital structural penile malformations, ranking second only to cryptorchidism in male genital birth defects. The incidence of hypospadias is increasing, currently affecting approximately 1 in 200–250 individuals, and as a result it has attracted considerable attention within the medical community (2). Despite ongoing research, the exact cause of hypospadias remains unknown due to its complex pathogenesis and diverse clinical presentations. Some cases are associated with specific syndromes, which complicates both the study and treatment of the condition (3). Surgical intervention is the primary treatment for hypospadias, with approximately 300 surgical techniques available. However, regardless of the chosen method, postoperative complications are common, posing risks to the physical and mental health of affected children. As such, addressing how to minimize complications and improve surgical success rates is a critical issue in the medical field (4). Recent years have witnessed a surge in domestic and international research on hypospadias, leading to the expansion of academic literature and information overload. This abundance of data not only complicates the identification of current research trends in the field but also hampers new research initiatives, limiting the scope and depth of exploration. Given this intricate research landscape, streamlining the integration of information and identifying useful directions for research are essential for advancing hypospadias studies.

Bibliometrics, a method that involves the statistical analysis of published research to extract measurable data, illustrates how knowledge is utilized in publications, offers a comprehensive understanding of the research field, and sheds light on the evolution and changes within the research area. Bibliometrics also assists in identifying influential studies or scholars, providing researchers with a clearer perspective on research topics and uncovering the strengths and weaknesses of research. As a result, bibliometric analysis has gained popularity across various fields in recent years (5).

Fardod O'Kelly et al. have conducted bibliometric analyses of the top 150 highly cited publications on hypospadias. Doğan G et al. have also performed bibliometric analyses on the publications of hypospadias. Together, these studies have provided valuable insights into future research trends and have created new knowledge framework maps for researchers (6, 7). However, there remains a lack of comprehensive systematic analysis of the literature on hypospadias. With the increasing number of publications on this topic, it is crucial to systematically review the scientific knowledge structure within the literature, understand research trends, and identify hotspots in the field. Building on previous research, we conducted a bibliometric analysis of the literature on hypospadias from 1998–2023, with the aim of comprehensively assessing research trends and hotspots using the Web of Science Core Collection (WOSCC) database and tools such as CiteSpace, VOSviewer, and R-Bibliometrix. The analysis focused on the publication volume, citation frequency, research trends, major authors, research institutions, and keywords in the hypospadias literature. The ultimate goal of this research was to pinpoint research hotspots and forecast future development trends in the field.

## 2 Data source and search strategies

**Figure 1:**
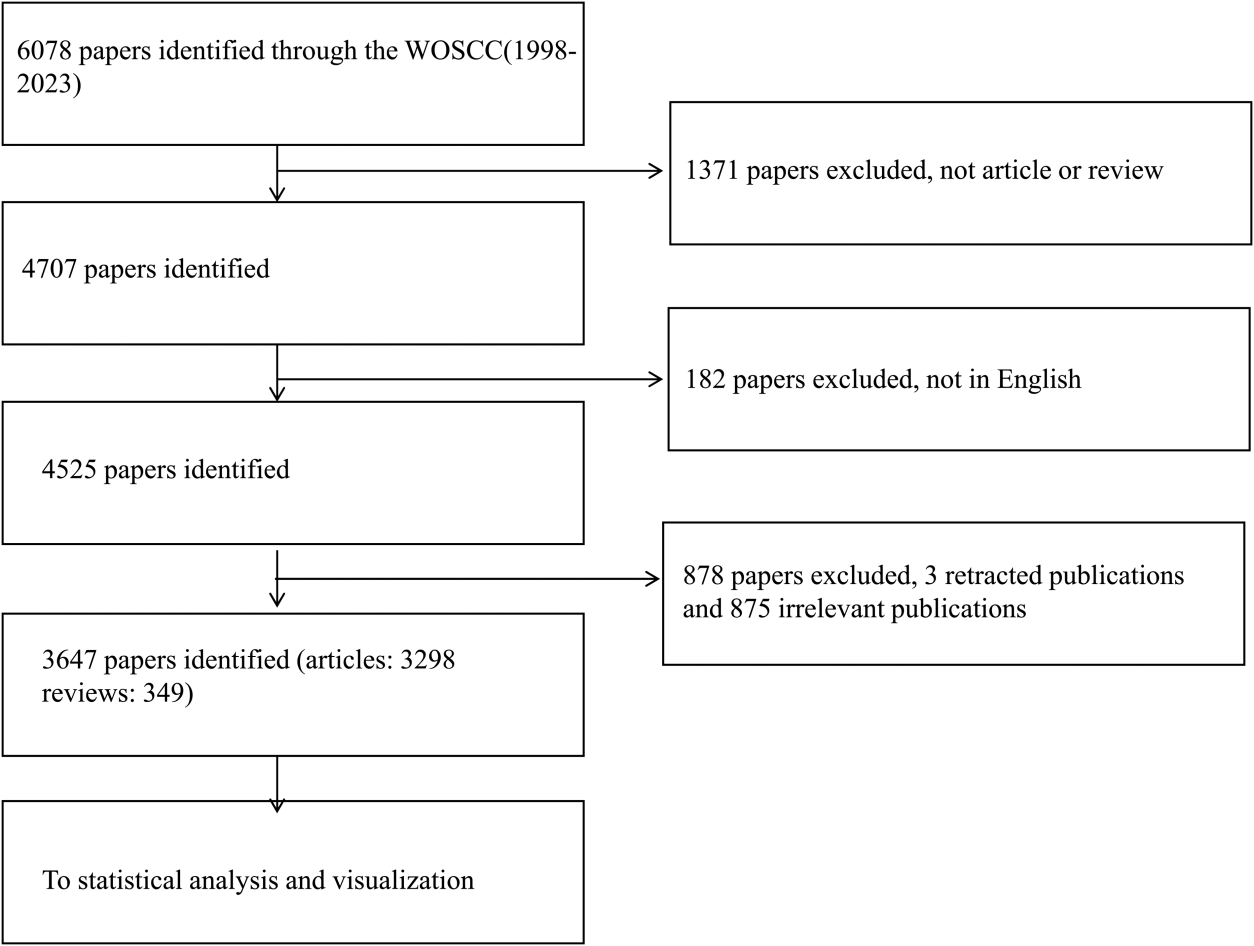
Data screening process.

For our database selection, we chose the widely recognized Web of Science for our bibliometric analysis. We conducted a comprehensive search of the WOSCC database for publications related to hypospadias from January 1, 1998, to December 31, 2023 (8). All searches were completed and downloaded on January 3, 2024, to prevent bias from daily database updates. Two authors independently verified the downloaded literature, and any disputed documents were resolved through full-text reading and discussions with corresponding authors. The search strategy used “hypospadias” as the search term, as the term “hypospadias mesh” was not found in PubMed. The inclusion criteria were limited to English-language articles or review articles. Initially, 6,078 documents were identified, with 4,525 meeting the criteria. After screening titles and abstracts, 3,647 articles that specifically focused on hypospadias were selected and imported into CiteSpace for deduplication, resulting in no duplicate documents being included, as outlined in Figure 1.

### 2.1 Method

In the current academic landscape, there is a range of bibliometric analysis visualization software available for scholars to utilize. For the present study, we opted to use CiteSpace, VOSviewer, and bibliometrix, all of which are well-established tools in the field, parameters were used for each method are summarized in Table 1. CiteSpace, created by Dr. Chen Chaomei from the Chinese Academy of Science and Technology, is specifically designed for document co-citation analysis. We used CiteSpace (version 6.2.R6) to conduct quality control measures, examine keyword prominence, and identify notable paper citations (8). VOSviewer (version 1.6.20) was employed to generate metrological maps based on co-occurrence data, providing visualizations in terms of networks, overlays, and densities. The visual map produced by VOSviewer uses nodes to represent various entities, including authors, journals, and keywords, with the node size indicating the frequency, line thickness denoting the connection strength, and the node color representing clusters or chronological aspects (9). We also employed R software and bibliometrix to monitor publication timelines and output levels of highly productive authors.

**Table 1:** Specific parameter settings for tool.

Tool	Analysis content	Parameter settings
CiteSpace	Literature quality control, keyword burst detection	Time slicing: 1998–2023, annual slices; Node type: keywords; Pruning methods: pathfinder, pruning sliced networks; g-index *k* = 25; Cluster analysis with default parameters, detection model configuration function: *f* (*x*) = ae−ax, ai−1ai = 2.0; The number of states: 2; *γ*[0,1] = 1.0; minimum duration: 2;
VOSviewer	Keyword clustering, journal co-citation, country publication, institution publication, author co-citation analysis	Co-occurrence threshold: ≥5 times; clustering resolution: 1.0; Visualization options: network, overlay, density
R-Bibliometrics	Author publication volume	Publication year and paper type

## 3 Results

### 3.1 Overview of publications

**Figure 2:**
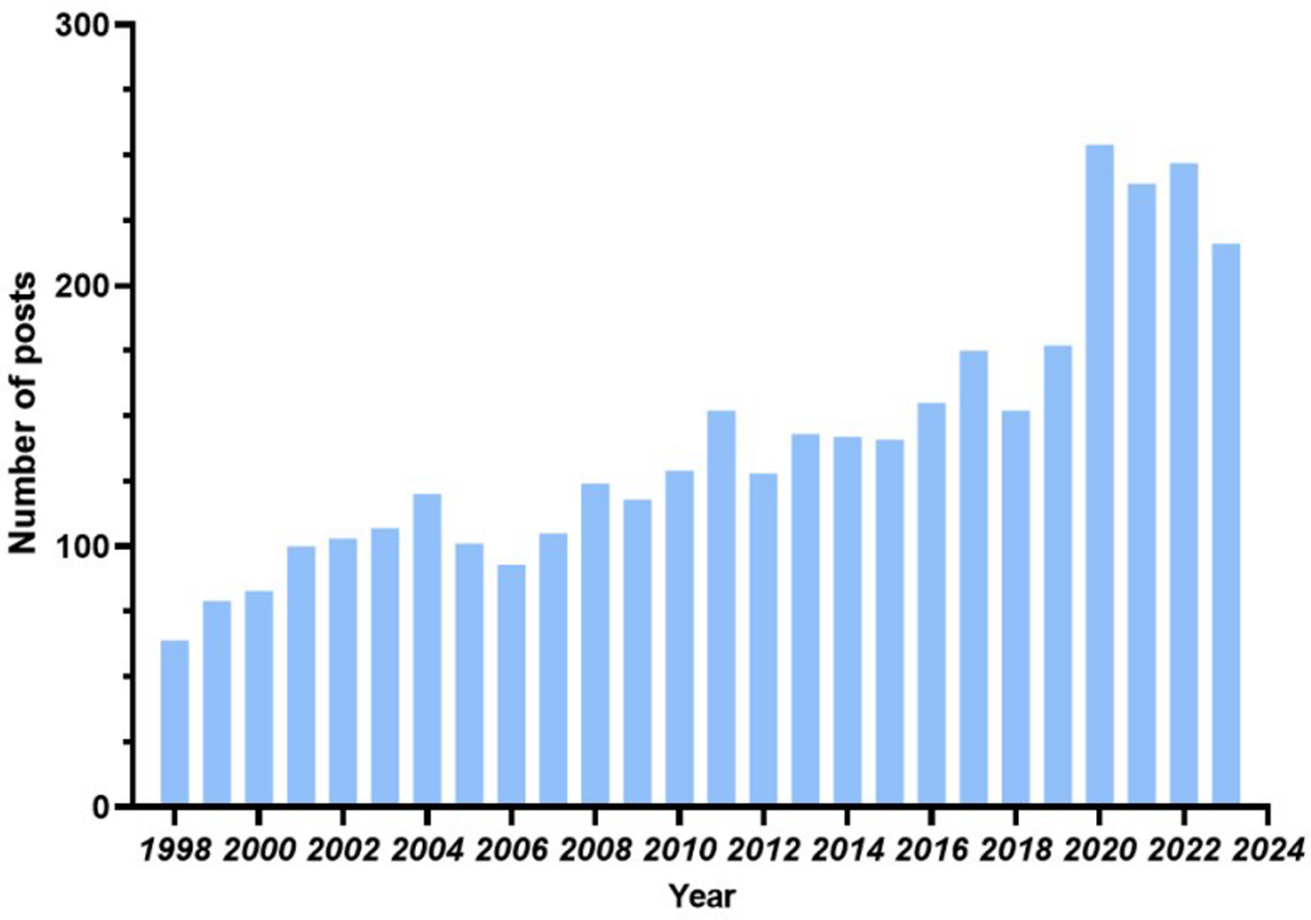
Annual publication.

The publication of scholarly articles is often regarded as a measure of advancement within a particular academic field (10). Fluctuations in publication rates can directly reflect shifts in the accumulation of scientific knowledge. The descriptive analysis was conducted using CiteSpace to examine the number of publications and growth trends. The study sample included 3,647 publications from 687 journals, authored by 14,633 individuals, representing 9,667 institutions across 487 countries. Among these publications, 3,298 were articles and 349 were reviews. The distribution of publications by year is illustrated in Figure 2. The annual publication count remained relatively stable from 1998–2016, indicating sustained interest in the field by scientists. However, there was a noticeable increase in annual publications from 2020–2023, exceeding 200, as well as a positive upward trend with consistent growth.

### 3.2 Author, institution, and country

#### 3.2.1 Author

**Figure 3:**
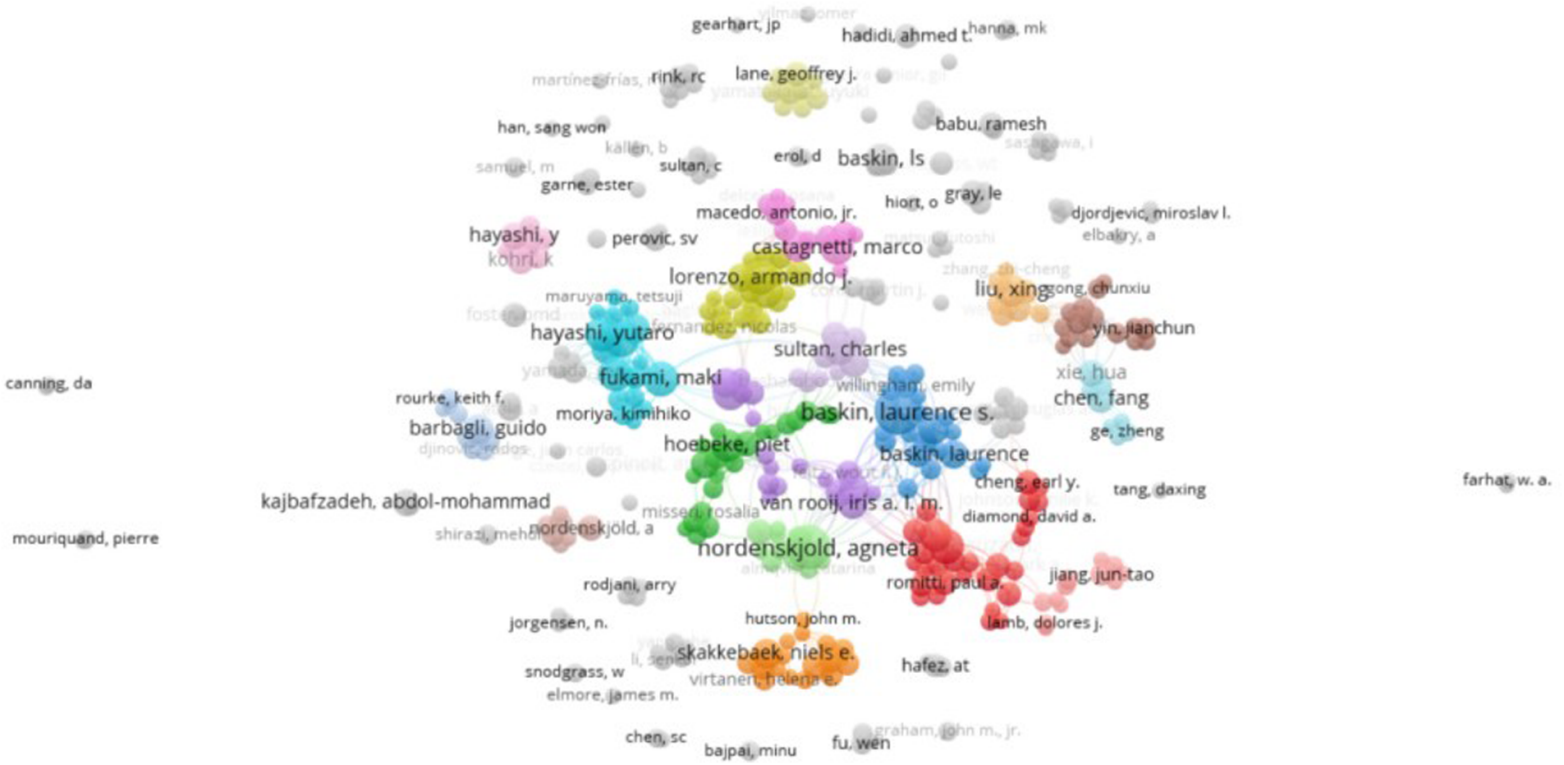
Author publication volume.

**Figure 4:**
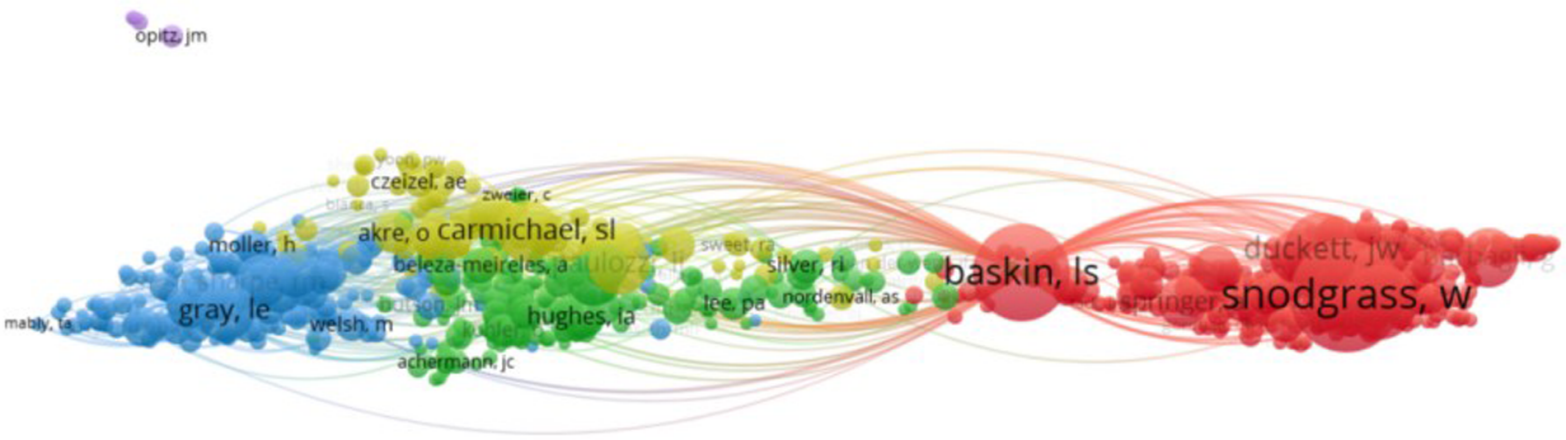
Author co-citation analysis.

A total of 14,633 authors were identified in the literature search, with an average of four authors per article. The authors' publication volume and co-citation data were analyzed and visualized using VOS. The top five authors with the highest publication volume and citation counts are summarized in Table 2. According to Price's law, the minimum number of core authors in a field is calculated as $m=0.749\times \sqrt{\mathrm{nmax}}$, where nmax represents the number of papers by the most prolific authors in the field. With nmax = 36 based on Citespace statistics, m is approximately 4.49. Therefore, authors who have published five or more papers are considered core authors in the field, totaling 370 individuals. The number of core authors with ≥5 papers is illustrated in Figure 3, while the number of co-citations reflects the authors' influence in the research field, as depicted in Figure 4 (11).

**Table 2:** The top five authors with the highest publication volume and citation counts.

Rank	Highest Publication Volume	Most Cited Authors
1	Baskin, Laurence S (38 articles)	Snodgrass, W (1,362 times)
2	Nordenskjold, Agneta (35 articles)	Baskin, LS (1,021 times)
3	Snodgrass, Warren (31 articles)	Duckett, JW (610 times)
4	Fukami, Maki (22 articles)	Carmichael, SL (407 times)
5	Hayashi, Yutaro (16 articles)	Paulozzi, LJ (407 times)

We conducted a brief review of the literature published by the top five cited authors. Baskin's research focuses on the anatomy and pathophysiology of hypospadias, as well as genetic research and advancements in surgical techniques. Nordenskjold's study emphasizes the genetics and molecular biology of hypospadias, including the identification and functional analysis of related genes. Fukami primarily investigates the molecular mechanisms of hypospadias, particularly the association between disorders of sexual development (DSD) and hypospadias. Snodgrass is renowned for developing and enhancing surgical techniques for hypospadias, notably the Snodgrass technique (also known as TIP). Hayashi delves into the surgical management of hypospadias and the assessment of long-term outcomes, encompassing postoperative complications and functional recovery (12–21).

Figure 4 illustrates the author collaboration analysis, showcasing 370 core authors who have published 5 or more papers, categorized into 74 author groups. Through an examination of the authors' citation network, 217 authors with more than 100 citations were grouped into six author clusters (Figure 4). The connections between circles represent collaborations among authors, with the circle size indicating the number of citations. The total link strength (TLS) metric reflects the impact of an author's published papers on other participating authors in the study. Notably, Baskin, Laurence S holds the highest TLS value (*n* = 1,645), followed by Nordenskjold, Agneta (*n* = 1,093).

**Figure 5:**
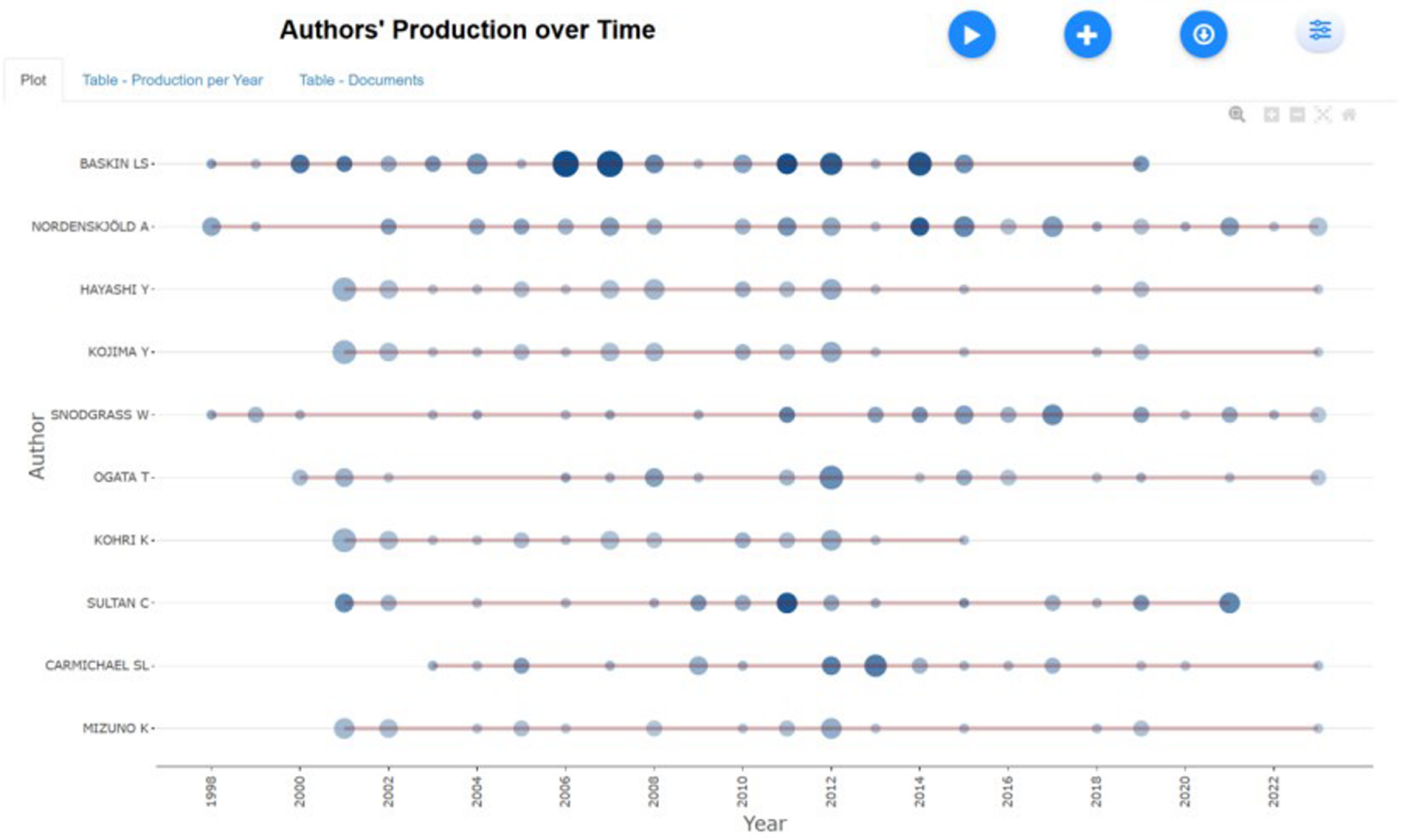
Publication volume and time distribution of prolific authors.

R software and bibliometrix were utilized to analyze the relationship between publication time and the quantity of publications by extremely productive authors within the particular research domain, as depicted in Figure 5.

#### 3.2.2 Institution

**Figure 6:**
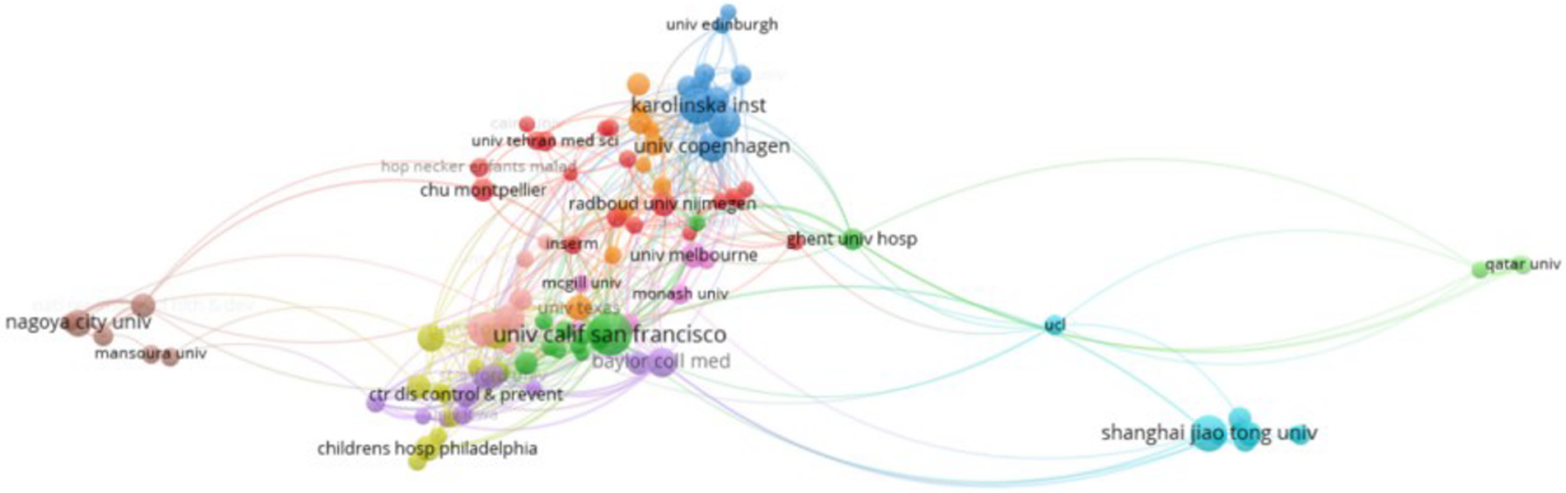
Institution publication volume.

The identified documents were published by 3,537 research institutions, with 148 institutions publishing more than ten articles. The top 10 institutions collectively published 541 documents, representing 14.8% of the total publications, as depicted in Figure 6. The top three institutions in terms of published documents were the University of California, San Francisco (*n* = 99), Shanghai Jiao Tong University (*n* = 67), and the Karolinska Institute (*n* = 63). Notably, Baskin, Laurence S is a prolific scholar at the University of California, San Francisco, while Agneta Nordenskjold is a prolific scholar at the Karolinska Institute.

#### 3.2.3 Country

**Figure 7:**
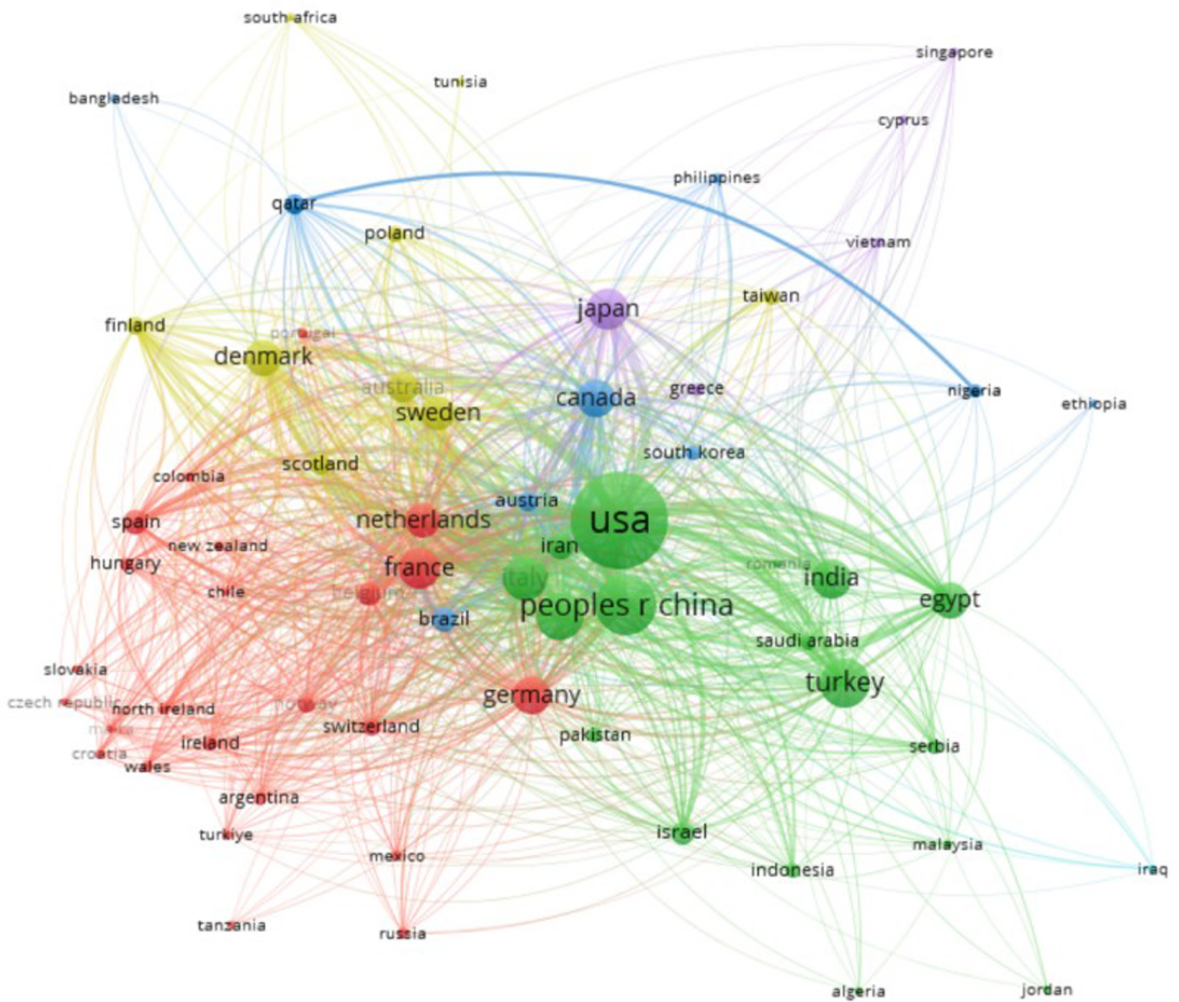
Country publication volume.

Based on the analysis conducted by VOSviewer, a total of 3,647 articles were collected from 112 countries. The top five countries/regions with the highest number of published articles, including the United States (*n* = 978, 26.8%), China (*n* = 382, 10.5%), Turkey (*n* = 257, 7%), the United Kingdom (*n* = 234, 6%), and Italy (*n* = 207, 5.7%). Together, these five countries accounted for 56% of the total publication count. Each node in the visualization represents a specific country or region, with the node size reflecting the number of published papers. The connections between nodes indicate collaborative efforts between countries, with a notable collaboration observed between the United Kingdom and the United States. The United States had a higher total link strength compared to other countries, suggesting a higher level of cooperation, as shown in Figure 7. This trend is supported by the greater number of articles from European and American countries in comparison to those from Asian, African, and Latin American nations.

### 3.3 Analysis of journals and co-cited academic journals

**Figure 8:**
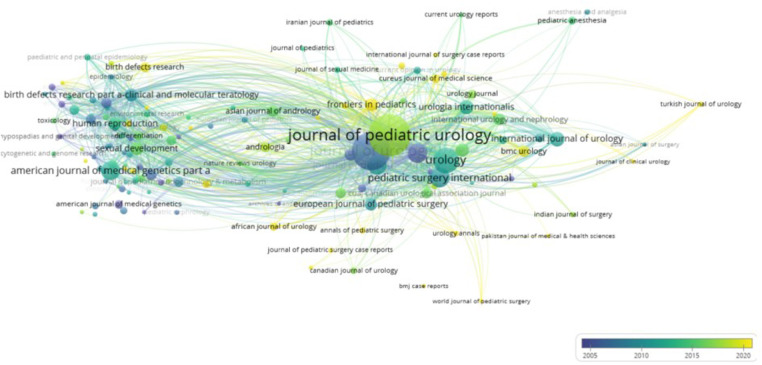
Journal co-citation analysis.

A total of 687 journals have collectively published 3,647 articles on hypospadias, with 134 journals contributing more than 5 articles each. Among the top five journals in terms of article count, the *Journal of Pediatric Urology* holds the first position, with 394 articles, followed by the *Journal of Urology,* with 330 articles, and *Urology*, with 148 articles. The top five most cited journals are detailed in Table 3. The impact of a journal is determined by the number of co-citations it receives, indicating its influence within a specific field. Notably, there are 13 journals that have garnered over 1,000 citations each. The most cited journal is the *Journal of Urology*, with 12,208 citations, followed by the *Journal of Pediatric Urology*, with 4,588 citations, as showed in Figure 8.

**Table 3:** Top five journals by publication volume.

Source	Documents	Citations	IF (2023)	JCR (2023)
Journal of Pediatric Urology	394	4,588	2	Q2
Journal of Urology	330	12,208	5.9	Q1
Urology	148	2,234	2.1	Q2
Pediatric Surgery International	92	993	1.5	Q2
BJU International	88	3,058	3.7	Q1

### 3.4 Keyword co-occurrence analysis

**Figure 9:**
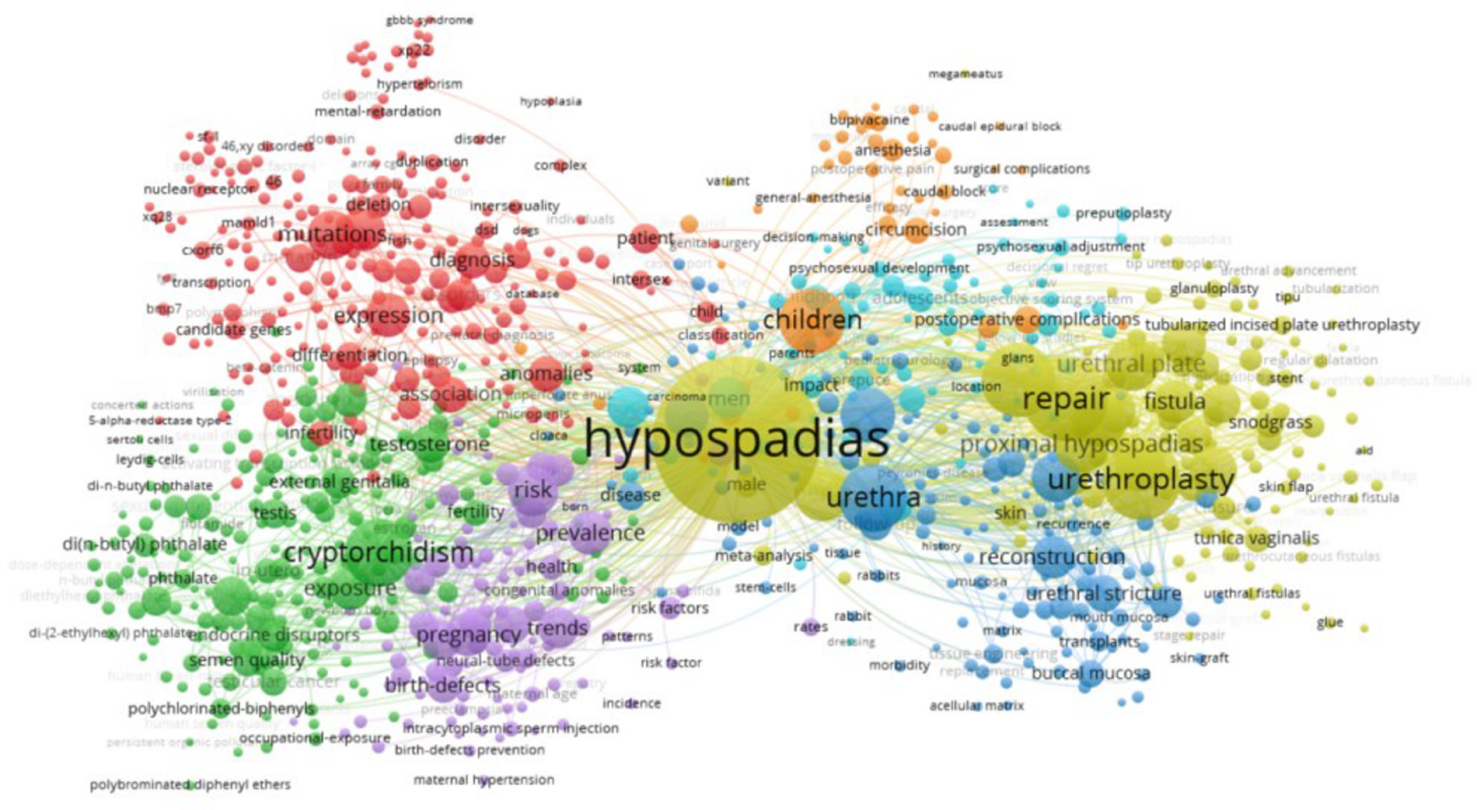
Keyword clustering analysis.

Keywords play a vital role in summarizing research content and pinpointing trends. To uncover research trends and popular topics, we selected 895 keywords that appeared at least six times out of a total of 8,856 keywords in the literature. These keywords were then analyzed for co-occurrence using VOSviewer. In the realm of network visualization, keyword cluster analysis is divided into seven distinct groups, as depicted in Figure 9. The largest cluster (red) encompasses 228 articles centered on analysis of the differential diagnosis, genetics, and molecular biology of hypospadias. This cluster explores topics such as “46, XY disorders of sex development,” “receptor gene,” and “srd5a2,” shedding light on the causes of sexual development abnormalities and genetic mechanisms behind hypospadias.

The second cluster (green), comprising 191 articles, delves into the connection between hypospadias and cryptorchidism within testicular hypoplasia syndrome, investigating environmental endocrinology through terms such as “testicular dysgenesis syndrome,” “androgen receptor,” and “endocrine-disrupting chemicals.” This cluster examines how external substances may impact androgen levels during embryonic development or disrupt the pathways leading to hypospadias.

The third cluster (blue), consisting of 135 articles, focuses on clinical research related to hypospadias, with keywords such as “anterior urethroplasty,” “buccal mucosal graft,” and “hypospadias complications.” This cluster evaluates the pros, cons, and potential complications of different surgical techniques and tissue types used in urethral contouring.

Cluster four (*n* = 123) (yellow) primarily focuses on clinical research related to the surgical treatment of hypospadias. This cluster is characterized by keywords such as “1-stage repair,” “dartos flap,” “dermal graft,” “granuloplasty,” and “incised-plate urethroplasty.” The research in this cluster explores the use of flap tissue from various sources to shape and cover the urethra, as well as the analysis of clinical outcomes of surgical interventions.

Cluster five (*n* = 101) (purple) delves into the impact of maternal and external factors on fetal birth defects, particularly within the genitourinary system, in children with hypospadias during the fetal period. Keywords such as “aetiology,” “birth defects,” and “assisted reproduction” are prevalent in this cluster, with evidence suggesting that the disease originates during embryonic development.

Cluster six (*n* = 74) (cyan) focuses on the study of complications following hypospadias surgery in adults, as well as parents' perspectives on childhood surgery for hypospadias. This cluster, containing keywords such as “adult hypospadias,” “decision making,” and “erectile function,” examines the evaluation of surgical outcomes in children with hypospadias by their families, long-term complications post-surgery, and the application of machine learning methods in hypospadias research.

Cluster seven (*n* = 43) (orange) primarily focuses on anesthesia-related topics in hypospadias surgery, with key terms including “anesthesia,” “caudal block,” and “neurotoxicity.” The research in this cluster investigates the impact of various anesthesia techniques on postoperative complications, pain management, and potential toxic effects in pediatric patients undergoing hypospadias surgery.

### 3.5 Analysis of development trends

**Figure 10:**
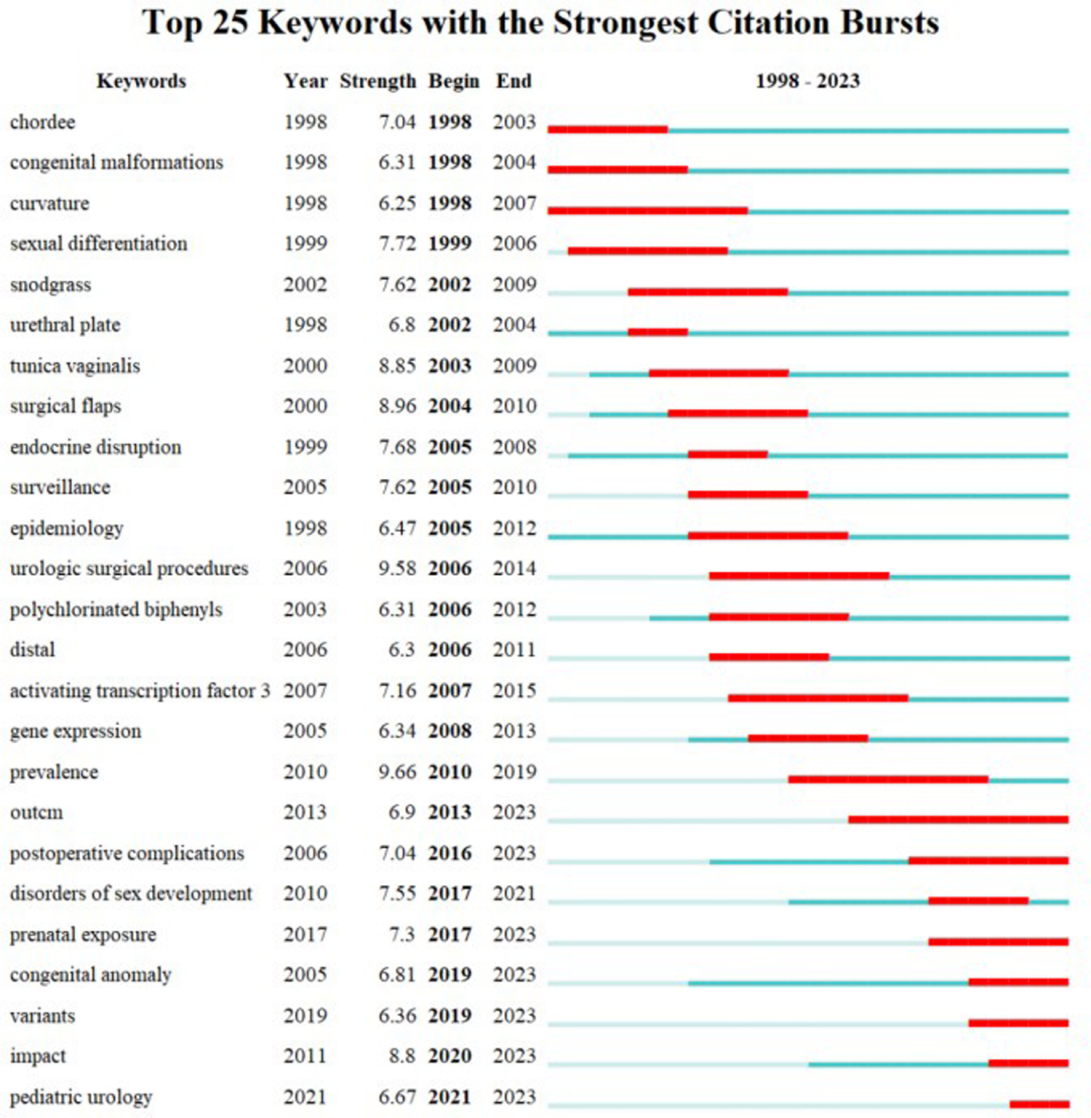
Keyword burst analysis.

In the analysis of keyword co-occurrence trends, researchers utilize time indicators to track shifts in research focus over a specific period. Visualizing keyword emergence shows that, in the past 2 decades, research on hypospadias has evolved from general clinical surgical methods to more precise flap selection and genomic studies. Advances in genetics and molecular biology have prompted a transition from enhancing surgical techniques to exploring pathogenesis, including gene identification, function, and environmental influences on hypospadias. Recent developments in gene editing, stem cell research, and tissue engineering have enabled a more precise investigation of hypospadias pathogenesis and the selection of optimal materials for urethral reconstruction, setting the stage for gene therapy and improved postoperative functional outcomes as in Figure 10.

## 4 Discussion

In this study, we delve into the historical evolution and advancement of hypospadias literature throughout different time periods. Prior to the 19th century, there were early descriptions and initial treatment attempts, with sporadic mentions in ancient medical texts but lacking systematic research and treatment approaches. Simple surgical procedures aimed to reconstruct the urethra, but faced challenges, with low success rates and numerous postoperative complications. From the late 19th century to the early 20th century, research primarily concentrated on improving surgical techniques, with a plethora of papers discussing the efficacy and enhancement methods of various surgical approaches. John W. Duckett's introduction of the “island flap” technique significantly enhanced surgical success rates and became the standard method in the mid to late 20th century (22). Subsequently, scholars compared different surgical techniques and explored strategies to prevent and manage postoperative complications. In the 1970s, “Meatal Advancement and Glanuloplasty” were proposed for mild hypospadias. In 1994, Warren Snodgrass developed the “tubular incision plate urethroplasty” (TIP), which is now widely utilized in various hypospadias repairs and is regarded as a cutting-edge technology in modern hypospadias surgery. Key research areas during this period included innovation and refinement of surgical techniques, as well as the management of complications (23).

By the beginning of the 21st century, advancements in genetics and molecular biology prompted a shift in research towards gene identification, functional studies, and the impact of environmental factors on hypospadias. Notable researchers, including Agneta Nordenskjold and Maki Fukami, investigated the genetic basis of hypospadias, highlighting the involvement of genes such as *CXorf6* in urethral development. Laurence S. Baskin and his team studied the effects of environmental toxins, such as endocrine disruptors, on hypospadias occurrence, revealing the complex interplay between genetics and the environment (24). In the 2010s, a surge in research focused on new technologies, personalized surgical approaches, regenerative medicine, and gene therapy for treating hypospadias. Innovative technologies such as 3D printing and high-resolution imaging, along with interdisciplinary collaboration, have allowed researchers to explore the anatomical complexities of hypospadias and develop personalized surgical strategies. Moreover, progress in stem cell and tissue engineering has advanced regenerative medicine for hypospadias, with ongoing studies on the use of stem cells to regenerate urinary tract tissues for improved function (25). Additionally, the emergence of gene editing tools such as CRISPR/Cas9 has provided researchers with new insights into the causes of hypospadias in animal models, highlighting the potential of gene therapy in this field (26).

## 5 Conclusion

This study has some limitations. First, the deadline for the analyzed publications was December 31, 2023, but the WOSCC data are continuously updated. Therefore, the gathered literature may not capture the latest developments in 2024, while the older literature may have incomplete data. Second, contributions from non-English literature may have been overlooked due to language selection restrictions. Despite these limitations, the study is significant as it systematically summarizes the knowledge structure and research frontiers of hypospadias research using bibliometrics and visual analysis. This report highlights new research hotspots and directions, indicating future breakthroughs in research and treatment with advancements in 3D printing, stem cell technology, and gene editing. Personalized treatment strategies targeting specific genetic variants are expected to improve efficacy and reduce complications. Furthermore, understanding the effects of environmental pollutants on sex hormone synthesis and signaling will lead to the development of public health strategies to mitigate exposure to endocrine disruptors, thereby improving the current scenario. Future research should focus on individualized treatment and effective prevention methods to enhance overall understanding of trends in the field.

## Data Availability

The raw data supporting the conclusions of this article will be made available by the authors, without undue reservation.
